# Brain temperature monitoring in newborn infants: Current methodologies and prospects

**DOI:** 10.3389/fped.2022.1008539

**Published:** 2022-10-04

**Authors:** Vinita Verma, Frederic Lange, Alan Bainbridge, Kelly Harvey-Jones, Nicola J. Robertson, Ilias Tachtsidis, Subhabrata Mitra

**Affiliations:** ^1^ Institute for Women’s Health, University College London, London, United Kingdom; ^2^ Medical Physics and Biomedical Engineering, University College London, London, United Kingdom; ^3^ Medical Physics and Engineering, University College London Hospital, London, United Kingdom

**Keywords:** brain temperature, neonatal seizure, neonatal encephalopathy, hypoxic ischaemic encephalopathy, cerebral metabolism, cerebral perfusion, near infrared spectrscopy (NIRS), magnetic resonance spectroscopy (MRS)

## Abstract

Brain tissue temperature is a dynamic balance between heat generation from metabolism, passive loss of energy to the environment, and thermoregulatory processes such as perfusion. Perinatal brain injuries, particularly neonatal encephalopathy, and seizures, have a significant impact on the metabolic and haemodynamic state of the developing brain, and thereby likely induce changes in brain temperature. In healthy newborn brains, brain temperature is higher than the core temperature. Magnetic resonance spectroscopy (MRS) has been used as a viable, non-invasive tool to measure temperature in the newborn brain with a reported accuracy of up to 0.2 degrees Celcius and a precision of 0.3 degrees Celcius. This measurement is based on the separation of chemical shifts between the temperature-sensitive water peaks and temperature-insensitive singlet metabolite peaks. MRS thermometry requires transport to an MRI scanner and a lengthy single-point measurement. Optical monitoring, using near infrared spectroscopy (NIRS), offers an alternative which overcomes this limitation in its ability to monitor newborn brain tissue temperature continuously at the cot side in real-time. Near infrared spectroscopy uses linear temperature-dependent changes in water absorption spectra in the near infrared range to estimate the tissue temperature. This review focuses on the currently available methodologies and their viability for accurate measurement, the potential benefits of monitoring newborn brain temperature in the neonatal intensive care unit, and the important challenges that still need to be addressed.

## Introduction

In term infants, neonatal encephalopathy (NE) is the most common form of perinatal brain injury in both high-income countries (HIC) as well as low- and middle-income countries (LMIC). Hypoxic-ischemic encephalopathy (HIE) affects 1–6 per 1,000 live births and remains a major cause of morbidity and mortality in term infants (1–3). Seizures are the most common presentation of neonatal neurological emergencies with an incidence of 1.9 to 2.2 per 1,000 live births (3). They are associated with further neuronal injury, poor neurological outcome, increased brain injury on MRI (4), and increased risk of epilepsy (5). Current research focuses on identifying optimal neuroprotective therapies for both these conditions to improve neurodevelopmental outcomes. A detailed understanding of the pathophysiological changes relating to brain metabolism and perfusion and their impact on brain tissue in real-time is a key factor in the assessment of both injury severity and its evolution.

Brain temperature is determined by the balance of heat production and heat removal, and it is influenced by multiple factors including cerebral metabolism, brain tissue injury, cerebral blood flow (CBF), body-brain temperature difference, various medications and infection (6–9). The brain and/or body temperature elevation is often associated with brain injury as seen in traumatic brain injury, adult stroke, and NE (10–13). The magnitude of temperature elevation has been shown to correlate with infarct size and severity in adults and is a risk factor for poor clinical outcomes (13–15). With significant changes in cerebral metabolism and perfusion following hypoxic-ischaemic injury and during neonatal seizures, it is likely to have brain temperature perturbation following perinatal brain injury and real-time brain temperature monitoring therefore might be an important biomarker.

## Changes in cerebral metabolism and perfusion during neonatal encephalopathy

The newborn brain suffers significant hemodynamic and metabolic derangements following hypoxic-ischaemic brain injury which causes a series of neurotoxic and neurochemical cascades over a period of several hours, days, and weeks post-injury (16). Early pre-clinical and clinical studies using phosphorous (^31^P) magnetic resonance spectroscopy (MRS) described the evolution of primary and secondary energy failure with a reduction in high energy phosphates and a rise in cerebral lactate following injury (17, 18). During the initial insult, a proportion of cells undergo primary cell death, and the neuronal supply of high energy metabolites such as adenosine triphosphate (ATP) is exhausted, also termed “primary energy failure.” Following successful resuscitation, the brain enters a latent phase lasting for ∼6–24 h which is characterized by the partial recovery of cerebral oxidative metabolism and cerebral blood flow, (CBF) although a degree of hypoperfusion continues (19, 20). The brain then enters a period of “secondary energy failure” (SEF) characterized by mitochondrial impairment and subsequent cell death with associated cerebral autoregulatory disturbance and brain hyperperfusion (21). Hypoxia-ischaemia induces significant cerebral inflammation with the production of proinflammatory cytokines (22) and activation of complement (23) that can further potentially be related to increased brain temperature. The concept of secondary energy failure (SEF) is a hallmark of NE and the primary target of current therapeutic hypothermia (TH) treatment. Abnormal autoregulatory control of cerebral haemodynamics with a combination of vasodilatation and vasoparalysis (24) is common after NE.

## Changes in cerebral metabolism and perfusion during neonatal seizures

NE remains the major etiological factor for the development of neonatal seizures and TH reduces the seizure burden following NE (25). Up to 75% of infants with NE can develop seizures (26) which are defined as transient symptoms of excessive or synchronous neuronal activity in the brain (27–29). Mitochondrial metabolism is closely related to neuronal activity. Studies using ^31^P MRS have revealed a drop in high-energy phosphates by one-third and an increase in mitochondrial oxidative phosphorylation by 45% during neonatal seizure (30), indicating a depleted cerebral energy state. Electroclinical and electrographic seizures produce an increase in cerebral blood flow velocity (CBFV) (31), likely to be due to excessive demand for glucose and oxygen but may still be insufficient to meet the pathological demands. A prolonged increase in cerebral blood flow is also likely to contribute to cerebral oedema and vasoparesis with accompanying loss of autoregulatory mechanisms. Cerebral autoregulation has been noted to be absent both during the seizures themselves and between seizures (32).

Previous studies with near-infrared spectroscopy (NIRS) during neonatal seizures have also described changes in cerebral hemodynamics and oxygenation (33–36). NIRS measures concentration changes in oxygenated [Δ(HbO_2_)] and deoxygenated haemoglobin [Δ(HHb)] which can then be used to derive changes in total haemoglobin [Δ(HbT)] and haemoglobin difference [Δ(HbD)]. Changes in [Δ(HbT)] and [Δ(HbD)] represent changes in cerebral blood volume and oxygenation respectively. In addition, cerebral oximeters can also measure absolute brain tissue saturation (or cerebral oxygenation) as an absolute percentage measurement of the mixed arterial and venous saturation. A seizure typically increases both cerebral blood flow and cerebral metabolic rate (37). Any increase in cerebral blood flow may not always be sufficient to match the cerebral metabolic demand during prolonged seizures, indicating cerebral blood flow-metabolism uncoupling (38) which can lead to reduced cerebral oxygenation. Previous studies in preterm infants suggested a 10% reduction in cerebral oxygenation to be of clinical concern (39), while animal studies using NIRS have found mixed cerebral oxygenation of 40%–50% (40, 41) to be the limits below which significant cerebral hypoxia with poor neurologic outcome occurs. Our previous work showed a rapid increase in the change in the oxidation state of cytochrome-c-oxidase [Δ(oxCCO)], noted at the onset of a seizure episode along with a rise in the baseline aEEG indicating an increase in neuronal activation and energy demand. Cytochrome-c-oxidase is the terminal electron acceptor in the mitochondrial electron transfer chain and is responsible for most of the ATP production during oxidative metabolism. Progressive decline in the [Δ(oxCCO)] baseline during seizures suggests a progressive decrease in mitochondrial oxidative metabolism (42). Neuronal energy demand rapidly increases at the onset of seizures reflected by a rapid increase in the mean aEEG activity coinciding with a rise in Δ[oxCCO].

## Methodologies for brain tissue temperature measurement

Several different methodologies have been evaluated so far for brain temperature measurements (Table 1). Proton MRS (43–50), microwave radiometry (51–53) and ultrasound thermometry (54). Other approaches are still under experimental evaluation in animals, for example, non-invasive wearable sensors to assess deep brain temperature based on skin thermal conductivity (55), or invasive optical fibre-based thermometry (56) Finally, there have been computationally based approaches to estimate brain temperature changes from traditional brain recordings such as MRI using mathematical models of brain temperature (57, 58) that consider the brain’s non-equilibrium thermodynamic nature between rest and functional activity. However, in clinical practice, these approaches remain to be tested. None of these methods is feasible for long-term ambulatory clinical use in newborn infants and requires large cost-intensive equipment. Optical methods for brain temperature measurement provide an option for use in clinical settings, even in ambulatory settings, given their ease of handling through their portability and cost-efficiency. Optical thermometry can furthermore, determine the efficacy of hypothermia, noninvasively and continuously throughout TH in HIE.

**Table 1:** Different methodologies for brain temperature monitoring.

	Non invasive	Continuous monitoring	Monitoring option in NICU	Portable	Cost-effective	Depth penetration	Ease of handling
MRS thermometry	Yes	No	No	No	No	Excellent	Poor
NIRS thermometry	Yes	Yes	Yes	Yes	Yes	Good	Excellent
Ultrasound thermometry	Yes	No	No	Yes	Yes	Good	Good
Microwave radiometry	Yes	No	No	No	Yes	Unknown	Good
Zero-heat flux sensor	Yes	No	No	No	Yes	Unknown	Poor
Invasive fibre-based optical thermometry	No	Yes	No	No	Yes	Good	Poor

## MRS brain thermometry

MRS thermometry has been used most for non-invasive and in-vivo brain temperature measurement in the neonatal population (47, 49, 59, 60). Clinical MRS reveals the prominent peaks of N-acetyl-aspartate (NAA), Choline (Cho) and Creatine (Cr). The much larger water peak is usually artificially suppressed as it obscures the visibility of the other spectral peaks. The relative position of a peak in the spectrum is described by its chemical shift. MRS thermometry is possible because of the temperature dependence of the water chemical shift relative to the temperature-insensitive chemical shifts of NAA, Cho and Cr. Careful measurement of the chemical shift separation between the water and reference-metabolite peaks, along with suitable calibration, can yield brain temperature estimation with an accuracy of ±0.5 °C and precision of 0.3 °C (59, 61, 62).

**Figure 1:**
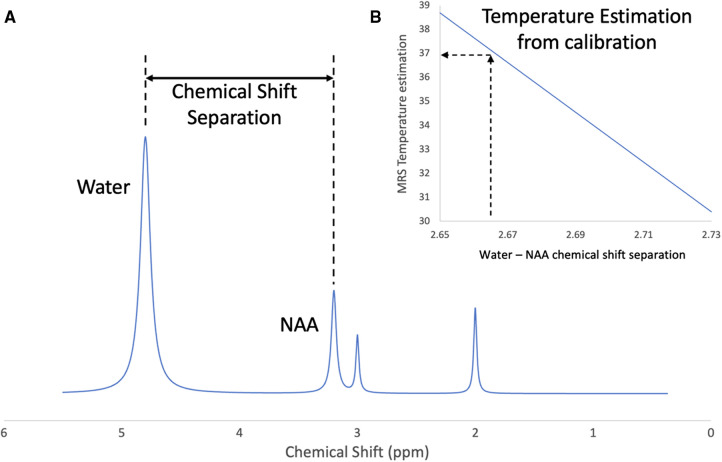
Brain temperature measurement using proton magnetic resonance spectroscopy. (**A**) Indicates the chemical shift separation between the water peak and a reference metabolite (NAA), (**B**) represents the calculation of brain temperature using a calibration based on the water-NAA chemical shift separation.

Non-invasive local temperature measurement using proton MRS has been demonstrated *in vivo* for many applications. The chemical shift of water is approximately linearly dependent on temperature in the physiological range (Hindman). So, by measuring the chemical shift separation between water and one or more reference metabolites, the absolute temperature can be inferred. Figure 1 describes the basic methodology. Spectra must first be obtained that show the un-suppressed water peak and the reference peaks. This can be done by acquiring a single un-suppressed spectrum and removing the water signal in post-processing to reveal the metabolite signals (50). Alternatively, this can be achieved by acquiring a water spectrum and a water-suppressed metabolite spectrum sequentially without changing the receiver frequency (49). By fitting an assumed line shape function to the water and metabolite peaks, the chemical shift separation in ppm can be measured and converted into temperature using an appropriate calibration.

## Challenges for MRS temperature measurement

The absolute change in the water peak chemical shift with temperature is very small, about 0.01 ppm per degree Celcius. Therefore, to measure temperature with a precision of 0.5 °C it is necessary to be able to measure the chemical shift separation to a precision of 0.005 pm. Compare this with a typical water linewidth *in vivo* of about 0.05 ppm. Despite this challenge, accuracies of 0.2–0.5 °C have been reported (59, 61–63).

### Choice of acquisition sequence

In-vivo MRS temperature measurement has been reported using both single voxel methods (49, 50, 59, 63, 64) and spectroscopic imaging (65–68). A challenge for both types of methods is obtaining a good shim to make the magnetic field as uniform as possible and thus minimise the water and metabolite linewidths. The movement of the subject during acquisition can make this even more challenging. Single voxel methods may be easier to implement to obtain a high precision measurement. In order to obtain sufficient signal to make the measurement, many repeated data acquisitions are averaged together. These can be compared and corrected for artefacts due to subject motion, resulting in an improved final measurement (50). However, single voxel acquisitions are limited to reporting temperature from a single location. Spectroscopic imaging methods allow the spatial variation of temperature to be mapped, but it can be more challenging to obtain a high-quality shim over an extended spatial region.

### Choice of calibrations and reference metabolite

The link between the measured chemical shift separation between the water and reference peaks, and temperature is the calibration data. Numerous calibrations have been published (59, 60, 69, 70). Changes in protein and ionic concentration have been shown to alter temperature calibration curves (71, 72). The apparent measured temperature decreases with ionic concentration by about 1 degree C per 100 mmol and increases with protein concentration (72). The calibration data, therefore, show a dependence on the conditions under which they were collected, and it is important to select an appropriate calibration for the desired application (73).

More than one reference peak can be used to make the temperature measurement. This can increase the precision of the measurement and the resilience of the measurement to pathological changes to the spectrum composition (59). Care must be taken when combining data from more than one reference peak so that the calibrations are internally consistent with each other (50). The choice of calibration will affect the level of systematic error in the absolute temperature measurement and so some care must be taken in interpreting MRS-derived temperature data. It is likely that temperature variation over time or relative spatial temperature differences are more reliable measurements than the absolute values themselves.

## NIRS brain thermometry

Optical methods based on NIRS can also be used to monitor tissue temperature. These methods are promising as they are based on safe and portable instruments that can be used to monitor the temperature continuously at the cot side. In the near-infrared region (650–1000 nm), the major endogenous tissue chromophores, responsible for the light’s absorption of the tissue, are water and oxy- and deoxy- haemoglobin. The aim of NIRS is to quantify the concentrations, or change in concentrations related to a specific event, of these chromophores in tissue and its focus is to quantify the oxygenation of the tissues. However, the linear temperature-dependent changes in NIR water absorption spectra make the measurement of tissue temperature possible with NIRS, as the tissues are mostly composed of water (more than 70% of the brain tissue composition for example).

**Figure 2:**
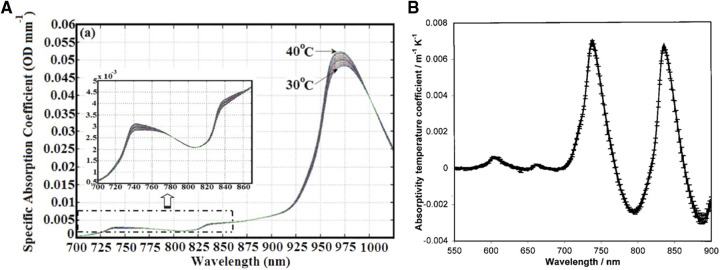
NIRS thermometry. Left (**A**) - NIR absorption spectra of pure water at various temperature. Extracted from Hollis et al. (74). Right (**B**) - Absorptivity temperature coefficients (da/dT) for the 550–900 nm. Extracted from Langford et al. (75).

Indeed, the water absorption spectra and its temperature dependency has been well established (74, 75). These spectra can be seen in Figure 2A (left), where the water absorption peaks shift to higher wavelengths and decrease in amplitude by approximately 0.8% per degree Celsius around 740, 840, and 970 nm with decreasing temperature (74). The absorptivity temperature coefficients between 550 and 900 nm can also be seen in Figure 2B (right) (extracted from reference 76), where the 2 peaks around 740 and 840 nm are clearly visible. Using this property, the temperature of the adult arm77 was measured using broadband continuous wave NIRS (BNIRS). In that paper, Hollis et al. (77) used the technique of principal component analysis (PCA) to calibrate the temperature response of the absorption spectra of pure water to predict the temperature response of the tissues. They particularly focused on the bands at 740 and 840 nm and reported a standard error of prediction of 1.2 °C. They noted that the results could be improved if the scattering properties of the tissues were accounted for, which was not the case in that model to retrieve the changes in temperature. A similar approach was then used by Holper et al. (78), focusing on the 840 band, in order to monitor the brain temperature of piglets and neonates. Using their methodologies, the authors reported an overall mean error bias between NIRS predicted brain temperature and body temperature of 0.436 ± 0.283 °C (animal dataset) and 0.162 ± 0.149 °C (human dataset).

Other studies using broadband diffuse optical spectroscopy (DOS) reported the measurement of the adult breas (79) and forearm (80). In these studies, the water peak at 970 nm was used to predict the temperature of tissues. The main limitation of this technique is that the strong absorption of the water at this wavelength limits its depth sensitivity, which makes it difficult to use for the monitoring of brain temperature in adults for example. However, the advantage of the use of DOS is to be able to disentangle the absorption and scattering properties of the tissues, thus reducing the impact of the scattering on the temperature measurement. Indeed, in NIRS measurements, as seen previously, it is often assumed that the changes in the optical signal came from a change in absorption only. However, in a clinical context, significant physiological changes can induce significant scattering changes which need to be considered to avoid crosstalk between scattering and absorption (81). Indeed, the scattering parameters of the tissue are mainly originating from the subcellular structures (82) but also from the cerebrospinal fluid layer in the subarachnoid space (83) and from the blood flow (84). Clinically, it has been shown that the resting scattering properties between the normal and affected areas in patients with traumatic brain injury (TBI) (85) and stroke (86) were significantly different. Moreover, dynamic changes in scattering are also detected when a large variation of blood flow is present (81). Thus, as blood flow variations are very likely in NE, changes in scattering coefficient can be expected. Therefore, one of the main challenges for NIRS instruments aiming at measuring brain temperature in a clinical context is to be able to measure both the absorption and scattering properties of the tissue in order to give accurate thermometry readings.

Finally, the brain temperature of piglets was measured using a time-domain NIRS (TD-NIRS) (87). TD-NIRS is known to be the most accurate of the NIRS techniques, can disentangle the absorption and scattering properties of tissues, and has the best depth sensitivity (88). Therefore, it makes it a great candidate to measure brain temperature even in difficult cases. Using this methodology, Bakhsheshi et al. used the bands at 740 and 840 nm in combination with the method of the PCA, introduced by Hollis et al., to monitor brain temperature in newborn piglets during cooling. The deep brain temperature (DBT) was also measured continuously with a thermocouple probe during this monitoring and ﻿the mean difference between the optical and DBT was 0.5 °C ± 1.6 °C. This methodology combined the two independent strengths of the studies previously mentioned: a scattering-free method (like DOS), increasing its accuracy, relying on the 740 and 840 nm bands (like BNIRS), and increasing its depth sensitivity.

Looking to the future, broadband TD-NIRS systems could further benefit brain temperature monitoring by NIRS techniques, as in general, the accuracy of the temperature prediction can be improved by acquiring a continuous absorption spectrum. Indeed, it allows a more accurate determination of chromophore concentrations compared to discrete wavelengths, as more chromophores can be quantified, and a more refined data analysis technique can be used. Such systems have been reported in the literature (89, 90), however, temperature monitoring was out of the scope of these studies. BNIRS is also able to monitor continuous absorption and scattering properties of light when appropriate algorithms and methodologies are used (91). Therefore, these 2 techniques appear to be good candidates in order to develop robust optical brain thermometry tools for clinical use.

The recent technological developments, notably in terms of electronics, enabled to reduce the footprint of TD-NIRS, which facilitated its use for clinical applications (88) and compact TD-NIRS systems are now available. Therefore, even though more work remains to be done in order to develop an accurate and robust optical instrument to monitor the brain temperature at the bedside, the recent technological developments make the possibility to develop small footprint instrument able to measure brain temperature now within reach. The next step will be to test the current methodologies in the clinic, as it has not been experimented so far.

## Discussion

Brain temperature is determined by the balance of heat production and heat removal, and it is influenced by multiple factors including cerebral metabolism, brain tissue injury, blood flow, body/brain temperature, drugs, sedation, seizures, and infection (6–9). The brain and/or body temperature elevation is often associated with brain injury as seen in traumatic brain injury, adult stroke, and HIE (10–13). The magnitude of temperature elevation correlates with infarct size and severity and is a risk factor for poor clinical outcomes in adults (13–15). MRS thermometry and mapping found the lowest body temperature at the core of tissue injury in adults with acute ischemic stroke and the highest brain temperature in the penumbral region (11).

Hypothermia decreases the metabolic demand for glucose and oxygen and attenuates secondary energy failure and neuroapoptosis (92, 93). The efficacy of TH depends on the target organ temperature and the neuroprotective effect depends on achieving the correct target temperature range (94, 95).

The current hypothermic strategy for NE uses rectal temperature for servo-controlled feedback to maintain a steady temperature profile. An in-vivo assessment of regional brain temperature using proton MRS during whole-body TH revealed heterogenicity of the brain temperature profile. Hypothermia effectively lower deep grey matter structures, whereas temperatures of more superficial structures in the grey matter and white matter are significantly greater than rectal temperatures (96). Findings also suggest that infants with MRI evidence of injury had overall higher and more homogenous brain temperature than those without injury (97). Non-homogenous patterns of brain temperature were also shown in other studies (50–66). In infants developing brain injury after NE, hypothermia decreased brain temperature during the first days of life but did not prevent an early increase of brain temperature (66). Wu et al. found significantly higher temperatures and brain-rectal temperature gradients in neonates with NE during TH (49). The application of using one specific baby temperature (33.5 °C) in NE for neuroprotection has improved the overall outcome of HIE but a single core temperature may not provide equal neuroprotective benefits to brain structures that have different histology, metabolic needs, and blood supply distribution. The threshold temperature to achieve neuroprotection may be unique to different brain structures. The heterogenicity of brain temperature while whole-body cooling raises the question of whether the current cooling system or methodology is the optimal way to cool. We need to explore whether there is a way to improve the homogeneity of white matter or cortical cooling and whether will this lead to improved neurological outcomes.

An animal study suggested that focal seizures produce an increase in neuronal activity and led to an elevation of local blood flow, cerebral metabolism and a significant rise in brain temperature (96). Generally, physiologic brain temperature is slightly warmer than core body temperature and subcortical structures are warmer than cortical structures (7, 67). However, injuries such as stroke can generate a brain-body temperature gradient, in which case core body temperature becomes a poor surrogate of brain temperature (98, 99). In adults with ischemic stroke, the temperature of the ipsilateral hemisphere is greater than the contralateral hemisphere (11, 61). Indeed, temperature alterations after neurologic injury, especially an increase in the brain or systemic temperature, are related to poor clinical outcomes (13, 15, 100, 101). During pathological processes such as neuroinflammation, increase metabolic demands overwhelm the brain’s already limited cooling mechanisms and drive temperature 1–2 °C higher than core body temperature (102).

In animal studies focal cooling rapidly terminates experimental neocortical seizures and histological examination of the cortex after cooling has shown no evidence of acute or delayed neuronal injury, and blood pressure and temperature remained stable (103). Animal experiments show that gentle cooling is capable of markedly reducing subsequent seizure frequency and intensity (104). The efficacy of TH in reducing seizure burden following NE has also been described (25).

Confirming brain injury at the bedside and determining the type and severity remains a challenge, as does bedside identification and monitoring of injuries likely involving ongoing processes of oxidative stress, excitotoxicity, inflammation, repair, and cell death, evolving over hours to weeks. As novel treatment strategies for neonatal brain injuries and seizures become available, the need for non-invasive and continuous bedside monitoring of disease severity and response to treatment becomes increasingly apparent. Non-invasive and continuous measurements of brain temperature in the neonatal neurocritical care set-up may permit the selection of neonatal candidates who may benefit from an adjustment in their hypothermia therapy or for additional neuroprotective therapies. Further studies are now urgently needed to establish whether optical brain monitoring can be a useful neuromonitoring tool in neonatal neurocritical care set-up. In view of the heterogeneous profile of brain temperature during TH, an option of continuous non-invasive monitoring of brain temperature at the bedside might also provide an opportunity to review and improve the current cooling methodologies in neonatal neurocritical care following NE.
